# The long non‐coding RNA SNHG1 promotes bladder cancer progression by interacting with miR‐143‐3p and EZH2

**DOI:** 10.1111/jcmm.15806

**Published:** 2020-09-04

**Authors:** Wei Xiang, Lei Lyu, Tao Huang, Fuxin Zheng, Jingdong Yuan, Chuanhua Zhang, Guosong Jiang

**Affiliations:** ^1^ Department of Urology Wuhan No. 1 Hospital Wuhan China; ^2^ Department of Urology Union Hospital Tongji Medical College Huazhong University of Science and Technology Wuhan China

**Keywords:** bladder cancer, CDH1, EZH2, HK2, miR‐143‐3p, SNHG1

## Abstract

The long non‐coding RNA (lncRNA) SNHG1 has been shown to be implicated in the progression of multiple human carcinomas. Nevertheless, the biological functions and potential mechanism of SNHG1 in bladder cancer (BC) are uncharacterized. In the present study, SNHG1 was found to be substantially up‐regulated in BC tissues and cells and was intimately correlated with the TNM stage, lymphatic invasion, metastasis and recurrence‐free survival in BC patients. Down‐regulation of SNHG1 dramatically attenuated the proliferation, migration and invasion of BC cells, whereas the ectopic overexpression of SNHG1 had the opposite effects in vitro. The in vivo experimental results also indicated that SNHG1 down‐regulation hampered the tumour growth and metastasis of BC cells. Mechanistic investigations revealed that SNHG1 enhances HK2 expression by serving as an endogenous sponge to regulate miR‐143‐3p in the cytoplasm of BC cells. In the nucleus, SNHG1 could interact with EZH2 and regulate the histone methylation of the CDH1 promoter, altering the biological behaviours of BC cells. Overall, these findings elucidate an oncologic role of SNHG1 in BC and provide a new therapeutic strategy against BC.

## 1 INTRODUCTION

Bladder cancer (BC), one of the most lethal neoplasms of the genitourinary system worldwide, has shown a trend towards increasing incidence and mortality rates in recent years.^1^ Despite substantial progress in understanding the pathophysiology of BC, the exact mechanisms underlying bladder carcinogenesis remain unclear. Therefore, identifying novel molecular targets and more effective therapeutic approaches is of crucial importance to improve the treatment and prognosis of BC.

Currently, emerging evidence has indicated that non‐coding RNAs (ncRNAs) are involved in the pathogenesis of human carcinomas, providing novel insights into mechanistic research on the occurrence and development of malignant neoplasms.^2^ miRNAs are a group of small ncRNAs with an average length of 20‐24 nucleotides that mediate the translational suppression or degradation of mRNAs by binding to their 3′‐untranslated regions (3′‐UTRs).^3^ Additionally, lncRNAs are a subgroup of ncRNAs that have lengths greater than 200 bp. It has been generally recognized that lncRNAs have key roles in the numerous processes, including epigenetic modification, transcription, post‐transcriptional modification and translation.^4^ Notably, increasing emphasis has been placed on mechanistic research of lncRNAs that affect the tumorigenesis and development of numerous human carcinomas, including BC.^1^, ^5^, ^6^ For example, the enhanced expression of lncRNA MALAT1 was characterized in BC and shown to promote metastasis by interacting with SUZ12.^7^ UCA1 was also observed to be up‐regulated in BC and has been identified as a sensitive and specific marker for BC.^8^ In addition, the aberrant expression of lncRNA H19 was demonstrated to be a risk marker for the development of non–muscle‐invasive BC.^9^ Other important lncRNAs such as BLACAT2, GAS5 and TUG1 have also been shown to be involved in the tumorigenesis and progression of BC.^10^, ^11^, ^12^ These findings demonstrated that lncRNAs are valuable diagnostic and prognostic biomarkers in BC. Nevertheless, further investigations are needed to elucidate the biological functions and underlying mechanisms by which lncRNAs influence the progression of BC.

Small nucleolar RNA host gene 1 (SNHG1) is an lncRNA located on chromosome 11q12.3. Recent reports have suggested that SNHG1 is aberrantly expressed and contributes to tumorigenesis in several types of human malignances, such as glioma,^13^ lung carcinoma,^14^ cholangiocarcinoma,^15^ breast cancer,^16^ hepatocarcinoma ^17^ and prostate cancer.^18^ Knockdown of SNHG1 in cancer cells induces their proliferation or growth and contributes to the diminishment of migration and invasion, indicating that SNHG1 has crucial roles in the maintenance of the above malignances. However, to our knowledge, whether SNHG1 has a strong impact on BC cell function remains unelucidated. In the present study, after demonstrating that SNHG1 up‐regulation is a characteristic feature of human BC, the biological role and downstream molecular mechanism of SNHG1 in BC progression were investigated. SNHG1 was ultimately identified as a competing endogenous RNA (ceRNA) that modulates the expression of HK2, which was further confirmed to be targeted by miR‐143‐3p in BC cells. Additionally, SNHG1 has been reported to bind histone methyltransferase EZH2, an essential enzymatic subunit of polycomb repressor complex 2 (PRC2), which catalyses the trimethylation of histone H3 lysine 27 (H3K27me3) in the promoters of target genes to inhibit target genes expression.^19^ The CDH1 gene encodes E‐cadherin, a well‐studied protein involved in maintaining the epithelial phenotype and modulating tissue homoeostasis by regulating various signalling pathways.^20^ E‐Cadherin has been characterized as a potent suppressor gene in numerous human carcinomas, including breast cancer,^21^ colorectal cancer,^22^ hepatocarcinoma ^23^ and BC.^24^ In the present study, SNHG1 was shown to recruit EZH2 protein to the promoter region of the CDH1 gene and epigenetically suppressed the expression of E‐cadherin in BC cells, modulating the biological behaviour of BC cells. Taken together, our results elucidated the mechanism associated with SNHG1 function in BC, indicating that SNHG1 can serve as an oncogenic marker in BC.

## 2 MATERIALS AND METHODS

### 2.1 Hierarchical clustering analysis and tissue sample collection

The Cancer Genome Atlas (TCGA) data were used to investigate the differences in SNHG1 expression between BC tissues (n = 414) and non‐tumour tissues (n = 19) using hierarchical cluster analysis. Sixty BC and paired non‐tumour tissue specimens were acquired from patients who received radical resection of tumour and were pathologically diagnosed as BC at Wuhan No. 1 Hospital, Tongji Medical College, Huazhong University of Science and Technology from March 2014 to October 2017. After surgery, the tissue samples were collected and kept in liquid nitrogen until analysis. The clinicopathological features of the patients are presented in Table 1. The patients were previously untreated, and all participants were asked to sign consent forms. The collection of clinical tissue samples was authorized by the ethical committee of Wuhan No.1 Hospital, Tongji Medical College, Huazhong University of Science and Technology, according to the ethical guidelines of the Declaration of Helsinki.

**Table 1:** Relationship between SNHG1 expression and clinicopathologic parameters of bladder cancer patients

Characteristics	Expression of SNHG1 in bladder cancer			
	Number of cases	Low	High	*P*‐value
Overall	60	n = 14	n = 46	
Gender				
Male	45	11	34	.7245
Female	15	3	12	
Age (y)				
<55	13	4	9	.4739
≥55	47	10	37	
Tumour size (cm)				
<3	17	9	8	**.0007** *
≥3	43	5	38	
Histologic grade				
Low	17	5	12	.4840
High	43	9	34	
Pathological stage				
pTa‐T1	11	6	5	**.0068** *
pT2‐T4	49	8	41	
Lymphatic invasion				
Negative	29	11	18	**.0097** *
Positive	31	3	28	
Distant metastasis				
Absent	38	13	25	**.0088** *
Present	22	1	21	

Bold values indicate significant difference.

* *P *< .05 was considered significant (Chi‐square test).

### 2.2 Cell culture

The BC‐derived cell lines (T24 and 5637) and the immortalized normal human uroepithelial cell line (SV‐HUC‐1) were acquired from the American Type Culture Collection (ATCC). The BC cell lines EJ and BIU‐87 were procured from the Shanghai Institute of Biochemistry and Cell Biology (Shanghai). All four BC cell lines were cultivated in a RPMI‐1640 medium (Invitrogen), and the SV‐HUC‐1 cells were maintained in F‐12K medium (Gibco) containing 10% foetal bovine serum (FBS, Gibco) and 1% penicillin/streptomycin (Gibco) at 37°C in a humidified incubator and under an atmosphere containing 5% CO_2_.

### 2.3 Subcellular fractionation assay

To isolate the cytosolic and nuclear fractions, the PARIS Kit (Invitrogen) was utilized. Then, the assay was performed based on the manufacturer's recommendation.

### 2.4 Quantitative reverse transcription PCR (RT‐qPCR)

Total RNA in BC samples and cell lines was harvested with TRIzol reagent (Invitrogen). For RT‐qPCR assays, 2 μg total RNA treated with DNase I was subjected to cDNA synthesis, with a PrimeScript RT‐polymerase kit (Takara). A StepOnePlus^TM^ RT‐qPCR System (Applied Biosystems) and SYBR Green (Takara) were used for RT‐qPCR analysis. The mRNA level of each gene was normalized to that of GAPDH. The quantity of miRNA was normalized to U6 expression. The specific primers for GAPDH, SNHG1, CDH1, HK2 and other genes were obtained from Sangon (Shanghai, China), while those for miRNAs and U6 were acquired from RiboBio (Guangzhou, China). The relative expression of each gene was assessed using the 2^−ΔΔCT^ method. The primers are shown in Table S1.

### 2.5 Cell transfection

Small interfering RNAs (siRNAs) against SNHG1, HK2, CDH1, and EZH2 and scrambled negative control siRNAs (si‐NC) were all designed and provided by RiboBio (Guangzhou, China), as were the miR‐143‐3p mimic, mimic NC, miR‐143‐3p inhibitor and inhibitor NC. The human SNHG1 cDNAs were inserted into the *BamH*I and *Age*I sites of the vector pcDNA3.1 (+) (Invitrogen). The short hairpin RNA vectors targeting SNHG1 (sh‐SNHG1) or negative control shRNA (sh‐NC) were procured from Genechem Co. (Shanghai, China). Transfection was conducted using Lipofectamine 2000 (Invitrogen), following the manufacturer's guidelines. The siRNA, shRNA and overexpression vector sequences are shown in Table S1.

### 2.6 Fluorescence in situ hybridization (FISH)

Fluorescence in situ hybridization (FISH) experiments were conducted to determine the subcellular location of SNHG1 in BC cells. SNHG1 antisense RNA probes labelled with fluorescein were obtained from GenePharma (Shanghai, China), and the sense RNA probe for SNHG1 was used as a control. The assays were conducted in accordance with the protocol.

### 2.7 Cell Counting Kit‐8 (CCK‐8) test, cell cycle analysis and 5‐ethynyl‐20‐deoxyuridine (EdU) assay

Cells were inoculated in 96‐well dishes at a density of 1.0 × 10^3^ cells/well, and then incubated for 24, 48, 72 and 96 hours. Tumour cell viability was evaluated by Cell Counting Kit‐8 (Sigma) following the manufacturer's instructions, and a spectrophotometer was available to record the absorbance at 450 nm. A flow cytometry test was performed to evaluate the cell cycle progression following the manufacturer's protocol (KeyGen Biotech.). Cell proliferation viability was assessed to determine cell viability using an EdU kit (RiboBio) after 48 hours transfection following the manufacturer's recommendations. Images were acquired and analysed using an Olympus microscope (Japan). Positive cells are indicated as red fluorescence as compared to the blue fluorescence of Hoechst 33342 in nuclear‐stained cells. Cell proliferation was assessed as the percentage of EdU‐positive cells.

### 2.8 Migration and invasion experiments

For migration testing, 4 × 10^5^ cells were cultivated in the top plate of the chamber of a 24‐well plate with an 8‐μm Transwell chamber and an uncoated membrane (BD Biosciences, USA). For Transwell invasion experiments, the Matrigel was diluted 1:3 in phosphate‐buffered saline (PBS) before being added to a 24‐well plate for polymerization at 37°C for 45 minutes. In these experiments, the indicated cells were harvested and incubated for 24 hours. Then, the migrated or invasive cells were fixed and stained with crystal violet, and at least three random fields were imaged.

### 2.9 Luciferase reporter assay

For luciferase analysis, the sequences of wild‐type or mutant SNHG1 were amplified and cloned into the luciferase vector pMIR‐Report. For miR‐143‐3p and HK2, the sequences of the luciferase vector 3′‐UTRs of wild‐type or mutant HK2 were generated and inserted into the pMIR‐Report. Subsequently, these plasmids and synthetic oligonucleotides were cotransfected into BC cells, and the cells were harvested 48h later. Relative luciferase activity was assessed with a Dual‐Luciferase Reporter Assay Detection kit (Promega).

### 2.10 Western blotting, haematoxylin‐eosin (HE) staining and immunohistochemistry (IHC)

Proteins from BC tissues and cells were isolated using RIPA buffer (Thermo Scientific). The collected proteins were then quantified using the bicinchoninic acid (BCA) method and subjected to Western blotting. Antibodies against HK2 (ab209847**)**, Ki‐67 (ab15580) and EZH2 (ab227648) antibodies were acquired from Abcam, while those against cyclin D1 (#2922), E‐cadherin (CDH1, #3195), vimentin (#3932), PCNA (#13110), MMP‐9 (#13667), GAPDH (D16H11) and p21 (#2947) were provided by Cell Signaling Technology, Inc. The secondary antibodies were obtained from the Wuhan Boster Biological Co. Haematoxylin‐eosin (HE) staining was performed to examine the xenograft tumour samples in nude mice models using standard methods. Immunohistochemistry (IHC) assays were performed to measure the protein levels of HK2, p21, PCNA, cyclin D1, E‐cadherin, vimentin, MMP‐9, EZH2, and Ki‐67 in rat xenograft tumour specimens or BC tissues, using the avidin‐biotin‐peroxidase complex (ABC) technique. Images were acquired with an Olympus microscope (Japan).

### 2.11 RNA immunoprecipitation (RIP) experiments

RNA immunoprecipitation (RIP) experiments were conducted with an EZ‐Magna RIP™ RNA‐Binding Protein Immunoprecipitation kit (Millipore). In brief, the BC cells were harvested and lysed in RIP lysis buffer according to the manufacturer's recommendations. Subsequently, pre‐incubated magnetic beads coated with indicated antibodies were immunoprecipitated with the supernatant of cell lysate for 6 hours at 4°C. Subsequently, the purified RNA was checked by RT‐qPCR.

### 2.12 Chromatin immunoprecipitation (ChIP) assay and RNA pull‐down experiment

Chromatin immunoprecipitation (ChIP) was performed with an EZ‐ChIP kit (Upstate Biotechnology, USA) following the manufacturer's instruction. Antibodies against EZH2 (ab191250) and H3 trimethyl Lys 27 (H3K27me3, ab192985) were purchased from Abcam. Premier Primer 5.0 was used to design the CDH1 promoter primers that bind adjacent to the transcriptional start site, and the ChIP primers were obtained from RiboBio. The primers used for the ChIP assay are available in Table S1. A Magnetic RNA‐Protein Pull‐Down kit (Pierce, USA) was utilized to conduct RNA pull‐down experiments following the manufacturer's protocol. The biotinylated RNA used to detect SNHG1 was obtained from RiboBio (Guangzhou).

### 2.13 Animal experiments

To investigate the effects of SNHG1 silencing on BC growth in vivo, EJ cells (6 × 10^6^) stably transfected with the sh‐NC or sh‐SNHG1 vectors were implanted into BALB/c nude mice (n* = *5 for each group). Five weeks later, the mice were killed, and tumour volume and weight were measured. IHC assays were performed to examine protein levels in xenograft tumour specimens. In another assay, the stably transfected cells (3 × 10^6^) were intravenously injected into the nude mice (5 weeks old, n = 5 per group). After 7 weeks, the mice were killed, and their lungs were dissected for further examination. All animal assays were approved by the Animal Care Committee of Tongji Medical College.

### 2.14 Statistical analysis

All statistical analyses were performed using GraphPad Prism 6.0 (La Jolla, USA). The data are expressed as the means ± SD. Student's *t* test, *Chi*‐square test, one‐way ANOVA and Spearman correlation analysis were applied to estimate the statistical differences among various groups, where *P* < .05 was considered significant.

## 3 RESULTS

### 3.1 SNHG1 expression is up‐regulated in BC and is associated with disease progression

**FIGURE 1:**
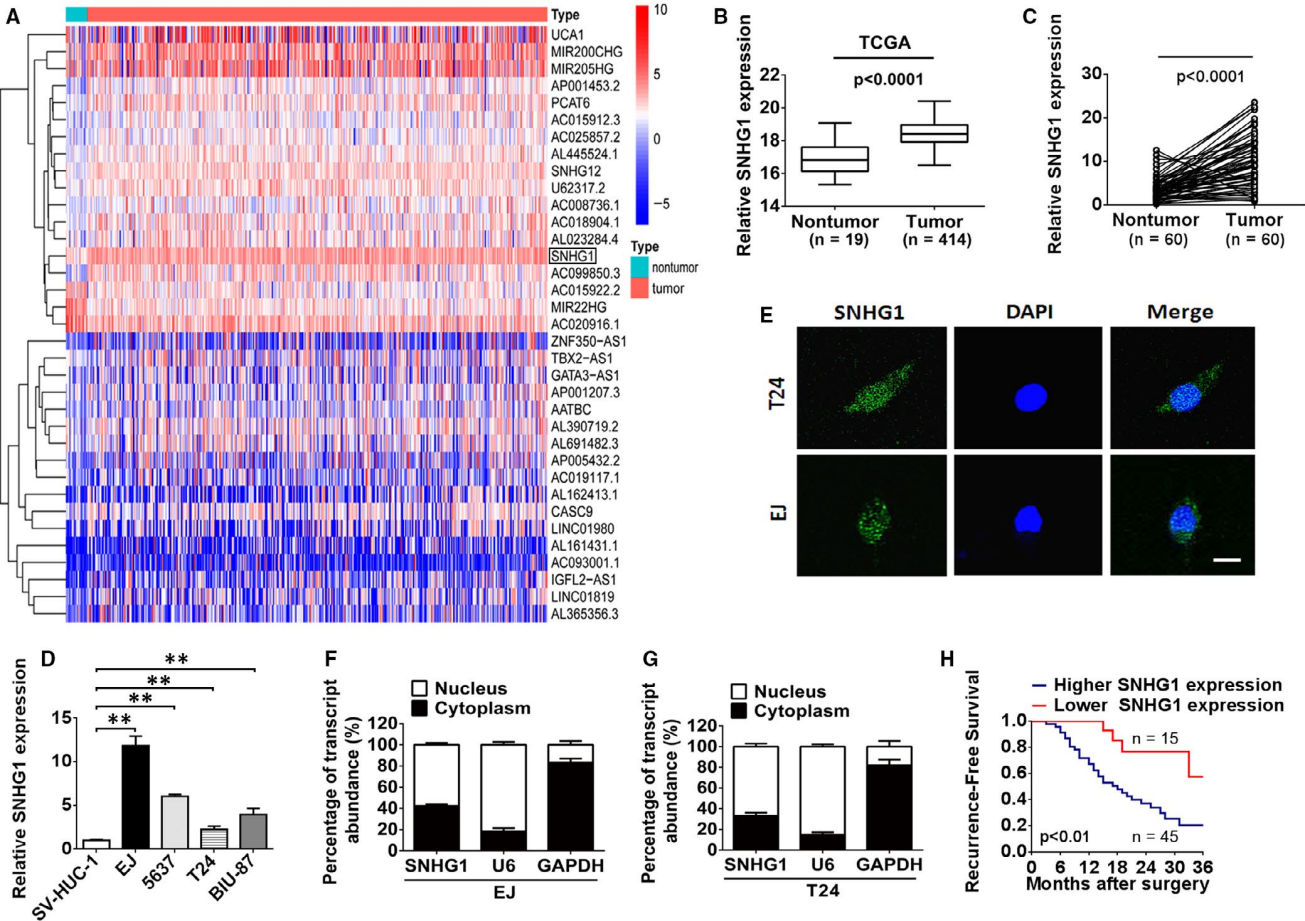
LncRNA SNHG1 expression is enhanced in BC tissues and cell lines. A, Hierarchical clustering heat map of lncRNAs that were differentially transcribed in BC tissues (n = 19) and non‐tumour counterparts (n = 414) from the TCGA dataset, with red indicating up‐regulation and blue indicating down‐regulation. The black box denotes SNHG1. B, The TCGA microarray datasets were analysed, and the expression of SNHG1 was observed to be remarkably enhanced in BC tissues (n = 414) compared with that observed in non‐tumour tissues (n = 19). C, Relative abundance of SNHG1 in 60 paired BC tissues and corresponding adjacent non‐tumour tissues. D, The level of SNHG1 expression was assessed in SV‐HUC‐1, EJ, T24, 5637 and BIU‐87 cells by RT‐qPCR. E, The subcellular location of SNHG1 in EJ and T24 cells (green staining) was checked by FISH analysis. DAPI was used for nuclear staining (blue). Scale bar, 10 µm. F‐G, After separation of the nuclear and cytoplasmic extracts, the relative RNA abundance was investigated via RT‐qPCR. U6 and GAPDH serve as nuclear and cytoplasmic markers, respectively. H, Kaplan‐Meier analysis of the recurrence‐free survival (RFS) in 60 BC patients. The 25th and 75th percentile expression levels of SNHG1 were treated as the cut‐off. ^**^ *P *< .01

An analysis of TCGA data (containing 19 normal bladder specimens and 414 BC specimens) revealed that the abundance of SNHG1 in the BC samples was higher than that observed in the non‐tumour bladder tissues (Figure 1A,B). Consistent expression was observed for the 60 paired BC specimens and non‐tumorous bladder tissues via RT‐qPCR. As shown in Figure 1C, SNHG1 expression was conspicuously enhanced in BC samples. When compared with the normal human uroepithelial cell line (SV‐HUC‐1), SNHG1 expression was significantly augmented in 4 different BC cell lines (EJ, 5637, T24 and BIU‐87) (Figure 1D). The results obtained using BC cell lines and BC tissues were consistent, further indicating that SNHG1 expression is increased in BC. The results of FISH and subcellular fractionation analyses revealed that SNHG1 is localized in both the nucleus and cytoplasm of BC cells, with a relatively high proportion of SNHG1 observed in the nucleus (Figure 1E‐G). In addition, the recurrence‐free survival (RFS) rate was determined through Kaplan‐Meier analysis. In the patient samples, SNHG1 expression was classified into lower SNHG1 and higher expression groups using the 25th percentile and 75th percentile expression levels as cut‐off points. A higher recurrence rate was observed in patients with high SNHG1 abundance compared with low SNHG1 expression (Figure 1H). Interestingly, the results of the clinicopathological analysis indicated that increased SNHG1 expression was closely correlated with the tumour size, tumour stage, invasion and metastasis (*P* < .05), whereas no significant correlation was observed between SNHG1 and other clinicopathological features, including sex, age and histologic grade (Table 1). We also investigated the expression levels of SNHG1 on the basis of clinical features using the TCGA databases. SNHG1 expression levels were increased in BC tissues compared with that observed in normal bladder tissues in the subgroup analyses, including tumour stage and nodal metastasis status (Figure S1A,B). However, no significant difference in the expression levels of SNHG1 was observed among different molecular subtypes (neuronal, basal, luminal, luminal infiltrated and luminal papillary) (Figure S1C). Taken together, these findings revealed that SNHG1 is most likely implicated in tumorigenesis and development of human BC.

### 3.2 SNHG1 promotes the proliferation, migration and invasion of BC cells

**FIGURE 2:**
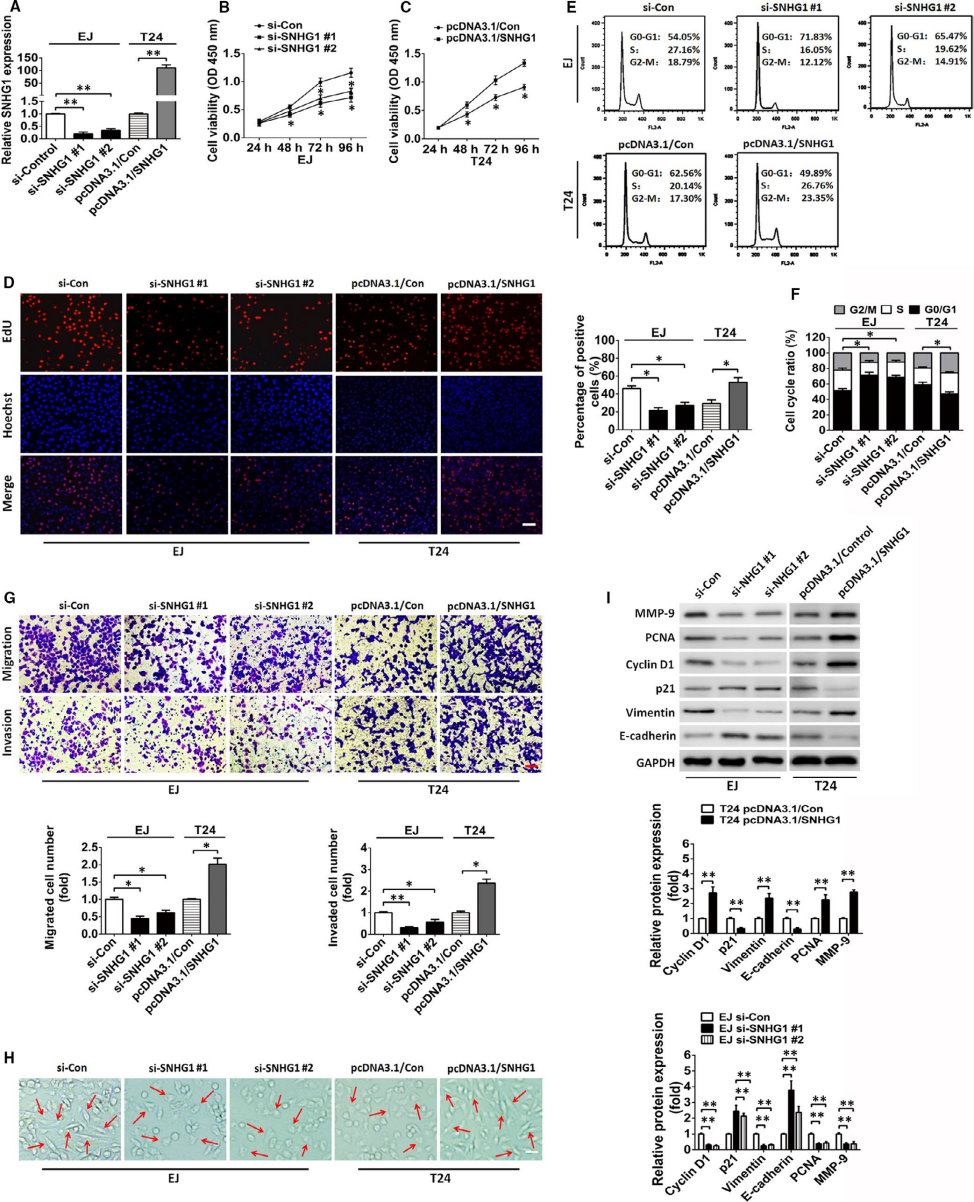
SNHG1 silencing suppresses the proliferation, retards cell cycle progression and inhibits the migration and invasion of BC cells in vitro. A, RT‐qPCR assay of SNHG1 levels in EJ and T24 cells following treatment with siRNAs against SNHG1 (si‐SNHG1 #1 and si‐SNHG1 #2) or an SNHG1‐overexpressing plasmid (pcDNA3.1/SNHG1). B, C, The proliferation of EJ and T24 cells transfected with si‐SNHG1 or pcDNA3.1/SNHG1 vectors were assessed via CCK‐8 analysis. D, The proliferation of EJ and T24 cells transfected with si‐SNHG1 or pcDNA3.1/SNHG1 were assayed by EdU assays. Scale bar, 50 μm. E, F, The effect of si‐SNHG1 or pcDNA3.1/SNHG1 on the cell‐cycle distribution of EJ and T24 cells was assessed by flow cytometry analysis. G, Representative images of the migratory and invasive BC cells transfected with si‐SNHG1 or pcDNA3.1/SNHG1 vectors are presented. Scale bar, 50 μm. H, EMT‐like morphological changes in EJ and T24 cells transfected with si‐SNHG1 or pcDNA3.1/SNHG1. Scale bar, 20 µm. I, The protein expression of cellular proliferation, migration, invasion and EMT‐related genes (p21, PCNA, cyclin D1, vimentin, E‐cadherin, and MMP‐9) was detected in si‐SNHG1‐ or pcDNA3.1/SNHG1‐transfected BC cells via Western blotting analysis. GAPDH served as an internal control. ^*^ *P *< .05 and ^**^ *P *< .01

As SNHG1 expression was observed to be substantially enhanced in BC, additional analyses were performed to determine whether it has an oncogenic effect in BC. First, we performed in vitro experiments to investigate the potential function of SNHG1. Among the four types of BC cell lines, a relatively high and low expression levels of SNHG1 were observed in EJ and T24 cells, respectively. Based on these results, the impact of SNHG1 knockdown or SNHG1 restoration in these cell lines was investigated. As shown in Figure 2A, SNHG1 expression was substantially reduced in EJ cells transfected with siRNA targeting SNHG1 (si‐SNHG1 #1 or #2) compared to that observed in cells transfected with the scrambled negative control (si‐Control). In contrast, SNHG1 expression was notably enhanced in T24 cells transfected with the pcDNA3.1/SNHG1 vector than in those transfected with the negative control. Subsequently, CCK‐8 and EdU assay results revealed that SNHG1 silencing markedly inhibited EJ cell proliferation, whereas SNHG1 overexpression facilitated the proliferation of T24 cells (Figure 2B‐D). Flow cytometry assay was performed to investigate whether SNHG1 affects BC cell proliferation by altering cell cycle distribution. As shown in Figure 2E,F, SNHG1 knockdown in EJ cells triggered cell cycle arrest in G0/G1 phase. As expected, overexpression of SNHG1 in T24 cells was shown to drive the entry of G0/G1 phase cells into S phase. Transwell chamber assays were conducted to evaluate the possible effect of SNHG1 on the migration and invasion of BC cells. As shown in Figure 2G, the decrease in SNHG1 expression by RNA silencing attenuated the migratory and invasive capacities of EJ cells compared with that observed in the negative control. In contrast, the ectopic overexpression of SNHG1 robustly accelerated the migration and invasion of T24 cells compared to that observed in the negative control. Epithelial‐mesenchymal transition (EMT) is a biological progress that enables a switch from an epithelial cell phenotype to a mesenchymal cell phenotype and is involved in tumour cell migration, invasion and metastasis. SNHG1 overexpression promoted an EMT‐like phenotype change in T24 cells, whereas SNHG1 knockdown exhibited an opposite effect in EJ cells (Figure 2H). These in vitro experimental results revealed that the deregulation of SNHG1 affects cellular activities, including cell proliferation, migration, invasion and EMT in BC. Moreover, Western blot analysis was used to detect the protein levels of potential proliferation/migration/invasion/EMT‐related markers within the biological process of BC cells. As shown in Figure 2I, SNHG1 silencing restored the protein levels of E‐cadherin and p21 but decreased that of PCNA, vimentin, cyclin D1 and MMP‐9 in EJ cells. In contrast, SNHG1 overexpression reduced the protein levels of E‐cadherin and p21 but elevated those of PCNA, vimentin, cyclin D1 and MMP‐9 in T24 cells. Notably, previous reports have demonstrated that CDKN1A (p21), PCNA, cyclin D1, vimentin, E‐cadherin and MMP‐9 are key factors involved in the modulation of tumour cell proliferation/migration/invasion/EMT in a variety of human cancers, including BC.^25^, ^26^, ^27^, ^28^, ^29^ We therefore predicted that these proteins may be useful markers to monitor biological behaviours of BC cells in SNHG1‐mediated modulation. Collectively, these results revealed that SNHG1 is capable of promoting BC progression by facilitating BC cell proliferation, migration, invasion and EMT.

### 3.3 SNHG1 silencing inhibits BC cell tumorigenesis and metastasis in vivo

**FIGURE 3:**
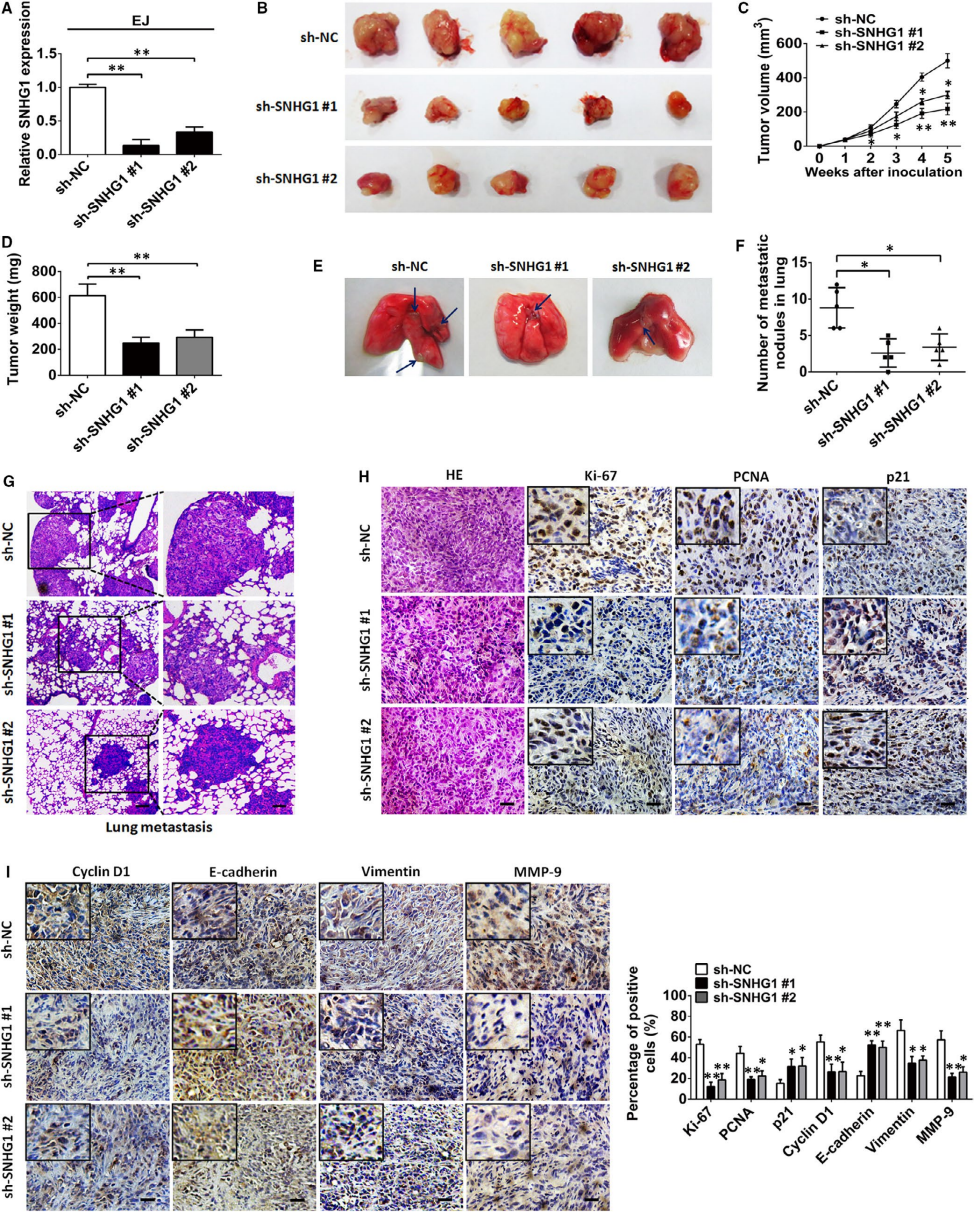
SNHG1 silencing suppresses tumour growth and metastasis in vivo. A, SNHG1 expression levels were assessed by RT‐qPCR in EJ cells harbouring a stable transfection with sh‐SNHG1 #1 or sh‐SNHG1 #2 vectors. B, Representative images of tumour xenografts in nude mice subcutaneously injected with sh‐SNHG1 #1‐ or sh‐SNHG1 #2‐transfected EJ cells after 4 weeks. Tumour volume (C) and tumour weight (D) were compared between the sh‐NC and sh‐SNHG1 groups. E, Representative sections of pulmonary metastatic mouse models are shown. The blue arrows indicate the tumour nodules. F, The number of metastases in the lung was assessed. G, Representative pathological images of the metastatic nodules stained by HE in the lungs. Scale bar indicates 200 μm in upper layer; 50 μm in lower layer. H‐I, Representative images of HE and immunochemistry staining for Ki‐67, p21, PCNA, cyclin D1, E‐cadherin, vimentin, and MMP‐9 in xenograft tumour tissues of mice in the SNHG1‐silenced group (sh‐SNHG1 #1 or sh‐SNHG1 #2) and the control group (sh‐NC). Percentage of positive marker expression of analysed marker genes was also assessed. Scale bar, 50 μm. ^*^ *P *< .05 and ^**^ *P *< .01

To elucidate whether SNHG1 influences the tumorigenesis and metastasis of BC in vivo, EJ cells stably transfected with sh‐NC vectors or sh‐SNHG1 plasmids (sh‐SNHG1 #1 and sh‐SNHG1 #2) were injected subcutaneously into nude mice. SNHG1 silencing remarkably inhibited BC cell growth in vivo and caused a significant suppression in tumour size and weight compared with that observed in the control group (sh‐NC) (Figure 3A‐D). Similarly, the number of lung metastatic nodules in pulmonary nude mouse model was reduced in the SNHG1 knockdown group compared with that observed in the control group (Figure 3E‐G). IHC analysis of xenograft tumour tissues revealed that SNHG1 silencing effectively reduced the level of Ki‐67 expression as compared with the control group. A lower Ki‐67‐positive rate was found in the sh‐SNHG1 group compared with that measured for the control group (Figure 3H‐I). Moreover, IHC analysis showed that SNHG1 silencing promoted the protein expression of E‐cadherin and p21 but inhibited that of PCNA, vimentin, cyclin D1 and MMP‐9 (Figure 3H‐I). Taken together, these results revealed that SNHG1 has an oncogenic role that can promote BC tumorigenesis and metastasis in vivo.

### 3.4 SNHG1 sponges miR‐143‐3p in the cytoplasm of BC cells

**FIGURE 4:**
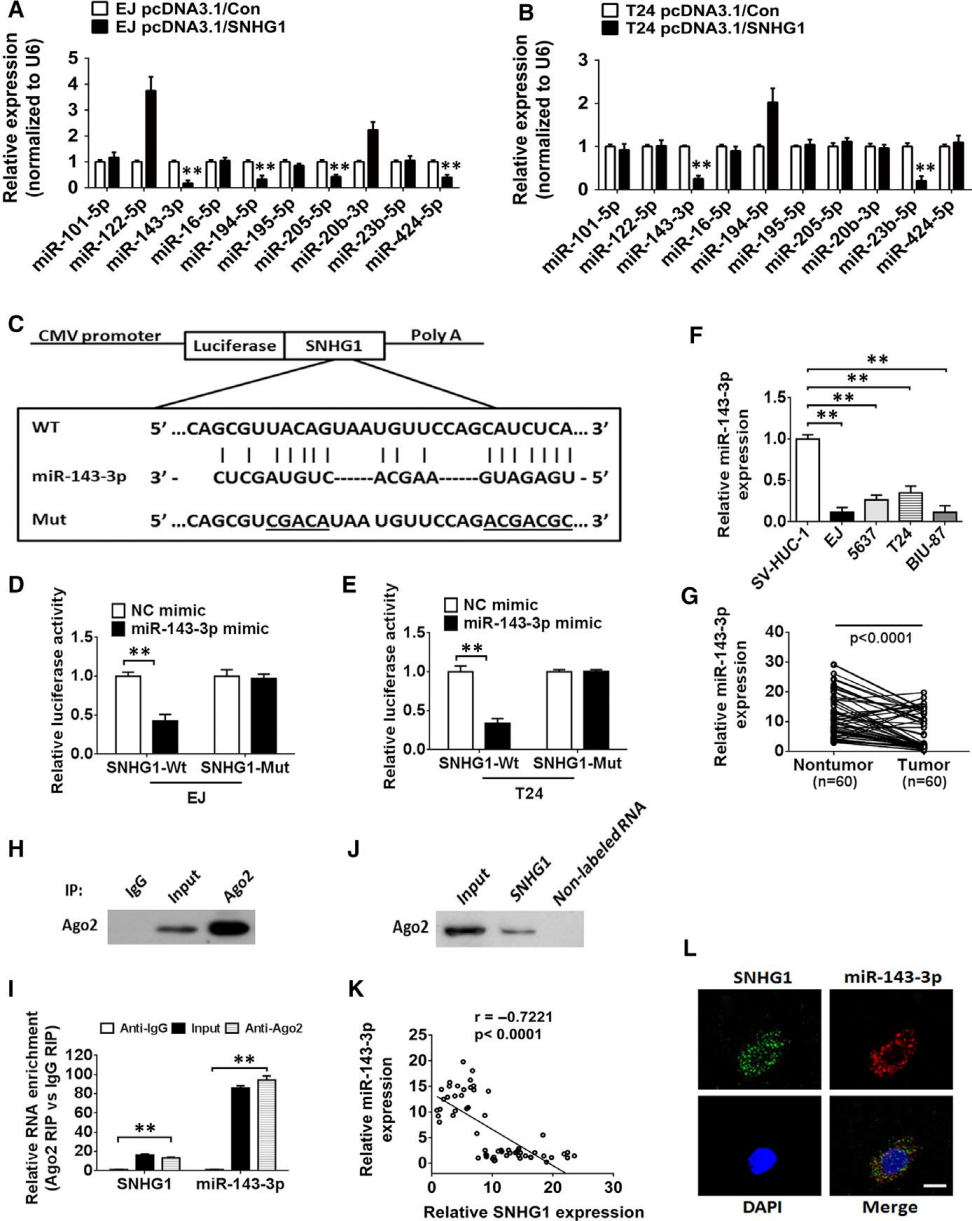
SNHG1 functions as an miR‐143‐3p sponge in the cytoplasm of BC cells. A, B, Relative expression levels of 10 candidate miRNAs in EJ and T24 cells transfected with pcDNA3.1/SNHG1 were determined by RT‐qPCR analysis. C, The putative binding sites for miR‐143‐3p in wild‐type/mutant SNHG1 transcripts. D, E, A luciferase activity assay was established to investigate the interaction between miR‐143‐3p and SNHG1 in EJ and T24 cells. F, The abundance of miR‐143‐3p in different BC cell lines was assessed via RT‐qPCR. U6 served as an internal control. G, RT‐qPCR analysis of miR‐143‐3p expression in 60 paired BC tissues and adjacent non‐tumour tissues. H‐I, A close correlation between SNHG1 and miR‐143‐3p was confirmed in EJ cell lysates by RNA immunoprecipitation (RIP) analysis. Upper panel: Ago2 analysis via IP‐Western blot analysis; lower panel: relative enrichment of specific RNAs in Ago2 or IgG immunoprecipitates. J, An RNA pull‐down experiment was conducted to assess the interaction between SNHG1 and Ago2 in EJ cells. K, An inverse correlation between SNHG1 and miR‐143‐3p expression was observed in paired BC tissues (n = 60, *r* = −0.7221, *P *< .0001). L, Representative FISH images showed the co‐localization of SNHG1 (green) and miR‐143‐3p (red) in the cytoplasm of EJ cells. Cell nuclei appear in blue (DAPI). ^**^ *P *< .01. Scale bar, 10 µm

Emerging evidence has confirmed that some lncRNAs can sponge miRNAs and regulate the expression and activity of targeted miRNAs.^30^ To determine whether SNHG1 has a similar regulatory mechanism in BC, LncBase v.2 was used to predict the potential miRNAs that may bind to SNHG1 (sheet 1 in Table S2). In addition, we searched for the down‐regulated miRNAs that have been identified as tumour suppressors in BC using an online server (http://mircancer.ecu.edu/) (sheet 2 in Table S2). Finally, 10 candidate miRNAs were selected by analysing the overlapping prediction results of miRNAs (sheet 3 in Table S2). As indicated in Figure 4A,B, miR‐143‐3p was the only miRNA that was remarkably down‐regulated by SNHG1 overexpression in both EJ and T24 cells. Subsequently, luciferase reporter cell lines were established to determine whether miR‐143‐3p was a functional target of SNHG1. The data indicated that the luciferase activity was obviously impaired in cells cotransfected with miR‐143‐3p mimics and wild‐type SNHG1 reporter vectors (SNHG1‐Wt) than in cells cotransfected with negative control (NC) mimics and SNHG1‐Wt in BC cells. Furthermore, these effects were blocked by mutating the putative miR‐143‐3p binding sites, suggesting that miR‐143‐3p was targeted by SNHG1 (Figure 4C‐E). Moreover, the abundance of miR‐143‐3p was observed to be markedly decreased in EJ, T24, 5637 and BIU‐87 cells compared with that observed in SV‐HUC‐1 cells (Figure 4F). As expected, the decreased expression of miR‐143‐3p was confirmed in BC tissues compared with that observed in non‐tumorous tissues (Figure 4G). We also investigated the potential biological function of miR‐143‐3p in BC cells. CCK‐8 and EdU assay results indicated that miR‐143‐3p overexpression suppressed the proliferation of EJ cells, while knockdown of miR‐143‐3p facilitated the proliferation of T24 cells (Figure S2A‐C). Flow cytometry results indicated that miR‐143‐3p overexpression triggered cell cycle arrest in G0/G1 phase in EJ cells, while miR‐143‐3p silencing promoted cell transition from G0/G1 to S phase in T24 cells (Figure S2D). These data revealed that SNHG1 targets miR‐143‐3p, which plays a tumour suppressive role in the modulation of proliferation in BC cells. Additionally, to further investigate whether SNHG1 directly interacts with miR‐143‐3p, RIP assays were conducted on EJ cell extracts. As shown in Figure 4H, Ago2 protein was obviously immunoprecipitated from the extracts of EJ cells using an anti‐Ago2 antibody. Furthermore, RT‐qPCR analysis of SNHG1 and miR‐143‐3p abundances in the immunoprecipitates showed a higher enrichment of SNHG1 and miR‐143‐3p in the Ago2 immunoprecipitate group compared with that in the control group (Figure 4I). SNHG1 was also demonstrated to interact with AGO2 protein in BC cells via RNA pull‐down assays (Figure 4J). Notably, SNHG1 was shown to have a negative association with miR‐143‐3p expression in BC tissues (*R* = −0.7221, *P* < .0001; Figure 4K). Furthermore, RNA FISH experiments indicated that SNHG1 and miR‐143‐3p were co‐localized in the cytoplasm of BC cells (Figure 4L). These results demonstrated that SNHG1 functions as a miR‐143‐3p sponge in BC.

### 3.5 HK2 is a direct target of miR‐143‐3p that is modulated by SNHG1

**FIGURE 5:**
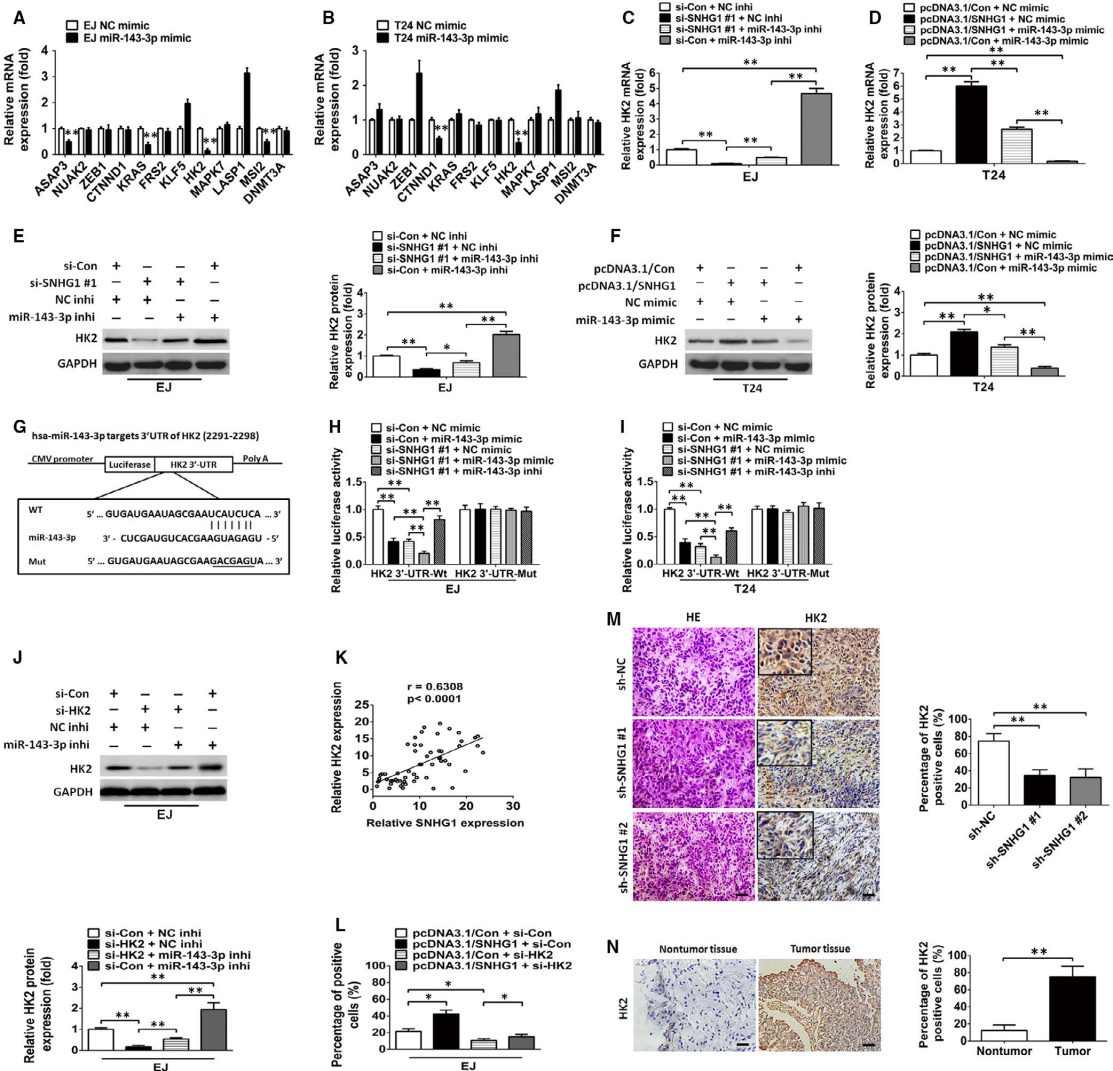
HK2 is targeted by SNHG1‐regulated miR‐143‐3p. A, B, RT‐qPCR analysis of mRNA expression levels of predicted miR‐143‐3p targets in miR‐143‐3p‐overexpressing EJ and T24 cells. C‐F, The levels of HK2 mRNA and protein were examined by RT‐qPCR and Western blotting analysis in cotransfected BC cells, respectively. G, The putative binding sites of miR‐143‐3p in the wild‐type/mutant HK2 mRNA 3′‐UTR sequence. H‐I, The luciferase activity assay results indicated that miR‐143‐3p overexpression or SNHG1 knockdown could remarkably inhibit the luciferase activity of the wild‐type HK2 3′‐UTR, but not the mutant HK2 3′‐UTR. And the decreased luciferase activity caused by SNHG1 silencing could be restored by miR‐143‐3p suppression in EJ and T24 cells. J, Western blotting analysis of HK2 protein expression in EJ cells following cotransfection with HK2 siRNAs and NC inhibitors or cotransfection with HK2 siRNAs and miR‐143‐3p inhibitors. K, A positive association was shown between the abundance of SNHG1 and HK2 mRNA (*r* = 0.6308, *P *< .0001). L, The cellular proliferation of EJ cells cotransfected with the SNHG1 overexpression vector and si‐Control or cotransfected with SNHG1 overexpression vector and HK2 siRNAs was assessed by EdU assays. M, IHC assessment of HK2 protein expression in xenograft tumour tissues. Scale bar, 50 μm. N, IHC assessment of HK2 protein in BC and paired non‐tumour bladder tissues. ^*^ *P *< .05 and ^**^ *P *< .01. Scale bar, 50 μm

As mentioned above, SNHG1 can sponge and inhibit the expression of miR‐143‐3p in BC. We next explored the underlying targets of miR‐143‐3p in BC. The potential targets of miR‐143‐3p were predicted using TargetScan and miRanda. A total of 197 genes were identified overlapping the prediction results from the two programs (Table S3). Then, we performed a targeted gene screen to identify potential miR‐143‐3p targets that may be associated with human cancer, resulting in 12 candidate genes (ASAP3, NUAK2, ZEB1, CTNND1, KRAS, FRS2, KLF5, HK2, MAPK7, LASP1, MSI2 and DNMT3A) being selected for further investigation. As indicated in Figure 5A,B, HK2 was shown to be markedly down‐regulated in both BC cell lines transfected with miR‐143‐3p mimics. Moreover, a reduction of miR‐143‐3p in SNHG1‐silenced cells alleviated the inhibition of HK2, while miR‐143‐3p overexpression restored the increase in HK2 expression in SNHG1‐overexpressed BC cells (Figure 5C,5D). Western blotting analysis also showed that the decreased protein expression of HK2, induced by SNHG1 silencing, could be reversed by the knockdown of miR‐143‐3p in EJ cells (Figure 5E). In contrast, the enhanced protein expression of HK2, induced by the overexpression of SNHG1, was further attenuated by the up‐regulation of miR‐143‐3p in BC cells (Figure 5F). Luciferase reporter analysis was performed to evaluate the bioinformatic predictions in which the pMIR luciferase reporter vectors containing either the wild‐type HK2 3′‐UTR (Wt) or a mutant sequence (Mut) (Figure 5G) were constructed and then cotransfected into BC cell lines with the miR‐143‐3p mimic, NC mimic, miR‐143‐3p inhibitor, si‐Control or si‐SNHG1. The data indicated that miR‐143‐3p overexpression or SNHG1 knockdown could remarkably suppress the luciferase activities of the wild‐type reporter group but not that of the mutant‐type HK2 3′‐UTR groups, and the repression of luciferase activity caused by SNHG1 inhibition could be reversed by miR‐143‐3p suppression (Figure 5H,I). Moreover, Western blotting results demonstrated that the increased level of HK2 protein in BC cells induced by miR‐143‐3p silencing was restored by cotransfection with si‐HK2 (Figure 5J). A positive association was observed between SNHG1 and HK2 in 60 BC tissues (Figure 5K). Notably, HK2 knockdown repressed SNHG1 overexpression‐mediated increase in BC cell proliferation (Figure 5L). IHC results indicated that HK2 expression was remarkably down‐regulated by SNHG1 knockdown in xenograft tumours formed from a stable EJ cell line (Figure 5M). Additionally, increased protein levels of HK2 were observed in BC specimens compared with those observed in adjacent non‐tumour tissues (Figure 5N). Taken together, these findings revealed that SNHG1 functions as a ceRNA to sponge miR‐143‐3p, restoring the capability of miR‐143‐3p to bind the 3′‐UTR of the target gene HK2 and inhibit its expression.

### 3.6 SNHG1 epigenetically silences CDH1 transcription via EZH2‐mediated H3K27 trimethylation

**FIGURE 6:**
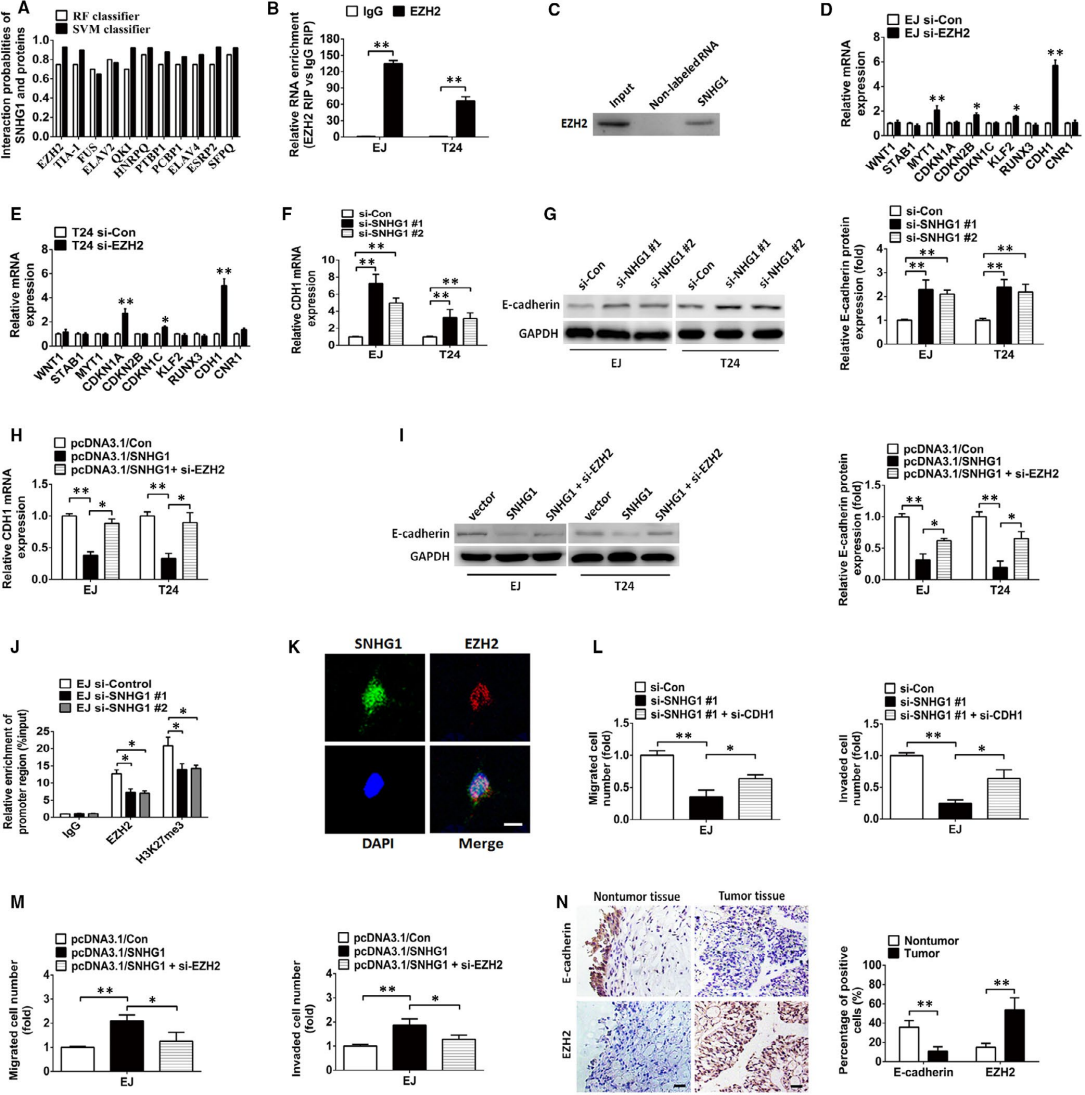
SNHG1 epigenetically inhibits CDH1 by interacting with EZH2 in the nucleus. A, The interaction probabilities of SNHG1 and potential binding proteins were estimated using a bioinformatics tool (http://pridb.gdcb.iastate.edu/RPISeq/index.html). B, RIP assays were performed and RT‐qPCR was used to assess the enrichment of SNHG1 in the co‐precipitated RNA. C, RNA pulldown was performed to detect the association of SNHG1 and EZH2. D‐E, RT‐qPCR analysis of mRNA expression levels of candidate genes in BC cells transfected with si‐EZH2. F, The abundance of CDH1 mRNA was evaluated in BC cells transfected with si‐Control or si‐SNHG1 via RT‐qPCR. H, RT‐qPCR assay of CDH1 mRNA level in BC cells transfected with the SNHG1 vector or cotransfected with the SNHG1 vector and EZH2 siRNAs. G‐I, Western blotting analysis of E‐cadherin under the indicated conditions. J, In ChIP experiments, RT‐qPCR was used to assess the occupancy of EZH2 and H3K27me3 in the CDH1 promoter region. K, Representative FISH images showing the co‐localization of SNHG1 (green) and EZH2 (red) in the nucleus of EJ cells. Cell nuclei were DAPI counterstained (blue). Scale bar, 10 µm. L, Cellular migration and invasion of EJ cells transfected with SNHG1 siRNAs or cotransfected with SNHG1 and CDH1 siRNAs were examined by Transwell assays. M, After transfection with SNHG1 vector or cotransfection with SNHG1 vector and EZH2 siRNAs, the migratory and invasive capabilities of EJ cells were assessed by Transwell assays. N, IHC analysis of E‐cadherin and EZH2 protein in BC and adjacent normal bladder specimens. Scale bar indicates 50 μm. ^*^ *P *< .05 and ^**^ *P *< .01

We also evaluated the potential functions of SNHG1 in the nucleus. A number of lncRNAs have been shown to bind proteins that regulate transcription and alter the expression of downstream genes.^5^, ^15^ We used the online RNA‐protein binding prediction site catRAPID (http://service.tartaglialab.com/page/catrapid_omics_group) to predict the ability of SNHG1 to bind to such proteins, and the results indicated that 579 proteins could potentially interact with SNHG1 (Table S4). Of note, previous reports revealed that lncRNAs located in the nucleus can recruit PRC2 to their target genes to regulate target genes expression.^31^ EZH2, the catalytic subunit of PRC2, mediates the trimethylation of histone H3K27, epigenetically repressing target genes in human cancers.^32^ Recent studies identified EZH2 as a non‐canonical RNA‐binding protein that is a key regulator in the development of tumours.^31^, ^33^ In the present study, the RNA‐protein interaction prediction tool (RPISeq, http://pridb.gdcb.iastate.edu/RPISeq/index.html) was also used to evaluate the potential interaction between SNHG1 and candidate RNA‐binding proteins. EZH2 and the top 10 predicted RNA binding proteins (Table S4 and Figure 6A) were subjected to further investigation through RIP assays, with the results indicating that only EZH2 could bind SNHG1 in both assayed BC cell lines (Figure 6B). Moreover, RNA pull‐down assay results also demonstrated that SNHG1 can specifically bind to EZH2 in BC cells (Figure 6C). We further detected the potential downstream targets of EZH2 in BC cell lines. Ten genes (WNT1, STAB1, MYT1, CDKN1A, CDKN1B, CDKN1C, KLF2, RUNX3, CDH1 and CNR1), which have been reported to be targeted by EZH2 in human tumours, were selected as candidate genes in BC. As shown in Figure 6D,E, CDH1 was demonstrated to be obviously up‐regulated by EZH2 knockdown in both BC cell lines. Subsequently, we investigated whether SNHG1 knockdown affected PRC2‐mediated epigenetic inhibition in BC cells. As shown in Figure 6F,H, RT‐qPCR results showed that SNHG1 silencing remarkably increased the mRNA expression of CDH1. In contrast, up‐regulation of SNHG1 resulted in an obvious decrease in CDH1 mRNA levels. However, the inhibitory effects were reversed by cotransfection with si‐EZH2 in BC cells. In accordance with the above results, those obtained by Western blotting indicated that down‐regulation of SNHG1 enhanced the protein expression of E‐cadherin, whereas restoration of SNHG1 hampered the expression of E‐cadherin. Notably, the reduced E‐cadherin protein expression induced by SNHG1 restoration was partially rescued by EZH2 knockdown (Figure 6G,I). We subsequently performed ChIP assays to investigate whether SNHG1 represses CDH1 transcription through the enrichment of H3K27me3 on the promoter region of CDH1. The data showed that SNHG1 silencing weakened the binding ability of EZH2 towards the CDH1 promoter (Figure 6J). Moreover, FISH assay disclosed that SNHG1 and EZH2 co‐localized in the nucleus of BC cells (Figure 6K). These results revealed that SNHG1 epigenetically represses the expression of CDH1 by binding to EZH2 in the nucleus of BC cells. To address whether CDH1 and EZH2 are implicated in the SNHG1‐mediated modulation of BC cell migration and invasion, we performed rescue assays. Transwell assays indicated that the silencing of CDH1 partially abolished the SNHG1 knockdown‐induced suppression of the migratory and invasive capabilities of BC cells (Figure 6L). Furthermore, EZH2 knockdown partially inhibited the effect of SNHG1 overexpression in promoting BC cell migration and invasion (Figure 6M). Additionally, IHC results indicated that E‐cadherin expression was reduced in BC tissues, whereas EZH2 expression was elevated in BC tissues (Figure 6N). Taken together, these findings illustrated the regulatory mechanism of SNHG1 in BC cell progression, by which SNHG1 functions as a platform for recruiting EZH2 to the promoter region of CDH1 that is epigenetically repressed by EZH2‐induced H3K27me3 formation.

## 4 DISCUSSION

The deregulation of oncogenes or tumour suppressor genes has been well demonstrated to drive cancer cell growth, invasion and metastasis,^34^ and increasing evidence has confirmed that lncRNAs are implicated in this process.^4^ In this study, lncRNA SNHG1 was shown to be remarkably up‐regulated in BC tissues and in different BC cell lines. Notably, several clinicopathological characteristics of BC patients, including tumour size, distant metastasis, lymphatic invasion, TNM stage and RFS were intimately correlated with higher SNHG1 expression levels, further suggesting that SNHG1 may be implicated in the pathogenesis and progression of BC. In the subsequent functional assays, consistent results were observed in loss‐ or gain‐of‐function analyses of SNHG1 in BC cells. The reduction in SNHG1 suppressed the proliferation, migration, invasion and EMT of BC cells, whereas the ectopic overexpression of SNHG1 had a negative effect on biological behaviours of BC cells. Strikingly, the in vivo experiments revealed that the silencing of SNHG1 significantly retarded the growth and metastasis of BC. These data showed that SNHG1 exhibits an oncogene‐like function in the modulation of BC properties.

Subsequently, the molecular mechanism by which SNHG1 silencing or overexpression modulated BC cell progression was further investigated. The functions of different lncRNAs have been generally recognized to be dependent on their subcellular localization. Nuclear lncRNAs have been shown to modulate target gene transcription or affect chromatin structure by recruiting related transcription factors or modification enzymes in the cell nucleus. In contrast, cytosolic lncRNAs have been shown to regulate the stability of mRNA, the localization of protein and to function as microRNA sponges.^35^ In the present study, SNHG1 was observed to be located in both the cytoplasm and nucleus of BC cells. Therefore, we further investigated the possible mechanism by which SNHG1 exerts its function in the BC cell cytoplasm and nucleus. Increasing evidence has shown that lncRNAs can post‐transcriptionally modulate the protein expression of miRNA target genes by sponging and inhibiting miRNAs in the cytoplasm.^30^, ^36^ For example, the lncRNA ARNILA was confirmed to sponge miR‐204 and to enhance breast cancer cell invasion and metastasis in breast cancer.^37^ It has been also demonstrated that lncRNA H19 sponges miR‐141 and contributes to tumour development and chemoresistance in human colorectal cancer.^38^ Interestingly, several groups have recently reported that SNHG1 functions as a key regulator that modulates the progression of various tumours by competitively binding miRNAs. For instance, Lu et al reported that SNHG1 suppresses the activity of miR‐145‐5p by binding to miR‐145‐5p in non‐small cell lung carcinoma.^14^ Another report revealed that SNHG1 sponges miR‐326 to facilitate the tumorigenesis of osteosarcoma.^39^ Moreover, Wang et al noted that SNHG1 promotes the progression of pituitary tumours by sponging miR‐302/372/373/520.^40^ In this study, we provided evidence that SNHG1 sponges miR‐143‐3p to disrupt its inhibition of HK2 in the cytoplasm of BC cells. Previous studies have revealed the tumour suppressor function of miR‐143‐3p in human carcinomas.^41^, ^42^ In accordance with a previous study,^43^ our present study confirmed that miR‐143‐3p expression was significantly decreased in BC tissues and cell lines. Subsequently, miR‐143‐3p was shown to directly target HK2, which exerts crucial functions in the modulation of cell proliferation. As a rate‐determining enzyme, HK2 catalyses the first essential step of glycolysis and is characterized as a metabolic hallmark of human carcinoma cells.^44^ The up‐regulation of HK2 has been reported in several types of human neoplasms, including BC, and the increased protein expression of HK2 was shown to be required for glucose metabolism and cancer cell progression.^45^, ^46^ Recent research has highlighted that some lncRNAs are involved in the reactivation of HK2 inhibited by miRNAs in human cancers.^47^, ^48^, ^49^, ^50^ Notably, we showed that SNHG1 acts as an endogenous ceRNA that regulates HK2 expression via sponging miR‐143‐3p in BC cells. In addition, SNHG1 expression was identified to be negatively associated with miR‐143‐3p expression, whereas SNHG1 expression was shown to be positively correlated with HK2 expression in BC specimens. These data were in line with our hypothesis and the previously reported findings,^51^ indicating the existence of a SNHG1/miR‐143‐3p/HK2 signalling axis in the cytoplasm of BC cells.

Evidence from a number of studies has implicated lncRNAs in the epigenetic modulation of gene expression via DNA methylation.^52^, ^53^, ^54^ The histone methyltransferase EZH2, the catalytic subunit of PRC2, has been confirmed to promote the trimethylation of H3K27 (H3K27me3), which is involved in the epigenetic silencing of target genes during cancer development. In our experiments, we showed that SNHG1 directly binds to EZH2 and is involved in the EZH2‐mediated epigenetic silencing of the EMT marker CDH1. As a member of the cadherin superfamily, CDH1 (E‐cadherin) expression is decreased and behaves as a tumour suppressor to block the migration, invasion and metastasis of numerous human carcinomas.^21^, ^22^ Additionally, the results of previous studies indicated that EZH2‐mediated hypermethylation of the CDH1 promoter induces the down‐regulation of CDH1 expression in human cancers, including BC.^55^, ^56^ In accordance with previous findings, our data confirmed that SNHG1 recruits EZH2 to the promoter region of CDH1 and then epigenetically suppresses the transcription of CDH1. Notably, the elevated levels of H3K27me3 induced by EZH2 were shown to be involved in this process. Moreover, the loss‐ and gain‐of‐function analysis results further proved that SNHG1 and the change in EZH2 and CDH1 expression are crucial in the modulation of BC cell migration, invasion, EMT and metastasis. Thus, in the nucleus, SNHG1 and its downstream target genes form a regulatory network that regulates the progression of BC.

**FIGURE 7:**
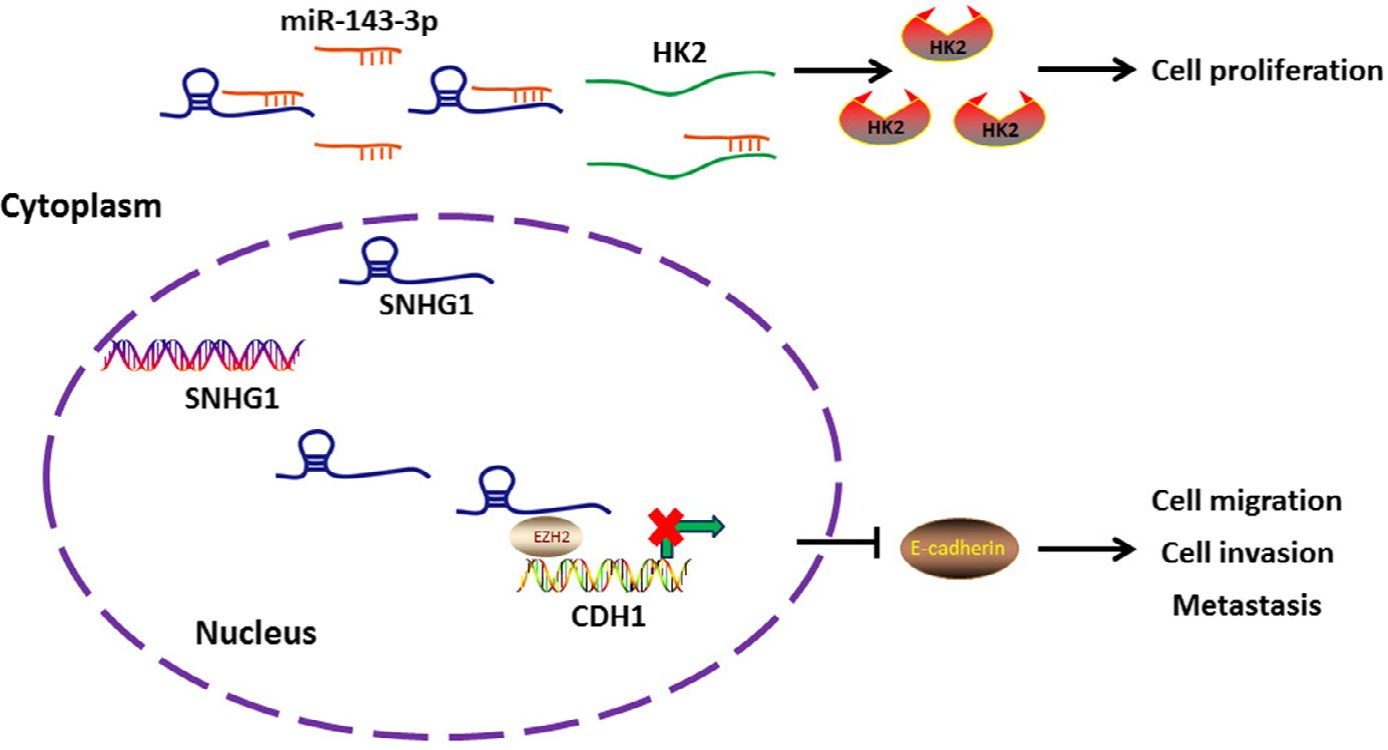
Schematic model of the proposed mechanism of SNHG1 in BC cells. SNHG1 has been confirmed to enhance the expression of HK2 by sponging miR‐143‐3p in the cytoplasm. SNHG1 has also been shown to be implicated in the EZH2‐mediated epigenetic suppression of CDH1 in the nucleus. The changes in HK2 and CDH1 expression is further implicated in the regulatory network which affects the biological processes of BC cells, including cellular proliferation, migration, invasion and metastasis

Taken together, the results of our present work highlights the oncogenic role of SNHG1 in human BC. In the cytoplasm of BC cells, SNHG1 sponges miR‐143‐3p to alleviate the inhibitory effects of miR‐143‐3p on HK2, thereby promoting the proliferation of BC cells. Additionally, SNHG1 was shown to interact with EZH2 and facilitates BC cell migration, invasion and metastasis via the epigenetic silencing of CDH1 in the nucleus (Figure 7). The present study not only reveals the diverse regulatory mechanisms of SNHG1 in different subcellular locations, but also represents a promising therapeutic option for inhibiting BC progression.

## CONFLICTS OF INTEREST

The authors confirm that there are no conflicts of interest.

## AUTHOR CONTRIBUTIONS

**Chuanhua Zhang:** Conceptualization (lead); Writing‐review and editing (lead). **Wei Xiang:** Investigation (equal). **Lei Lyu:** Investigation (equal). **Tao Huang:** Investigation (equal); Resources (equal). **Fuxin Zheng:** Resources (equal). **Jingdong Yuan:** Data curation (equal); Formal analysis (equal). **Guosong Jiang:** Data curation (equal); Formal analysis (equal).

## Supporting information

Fig S1

Fig S2

Table S1

Table S2

Table S3

Table S4

## Data Availability

All data generated or analysed during this study are included in this article.
