# Supplementary figures and images for: The long non‐coding RNA SNHG1 promotes bladder cancer progression by interacting with miR‐143‐3p and EZH2
Source: J Cell Mol Med. 2020 Sep 4;24(20):11858–73. doi: 10.1111/jcmm.15806 (PMC7578868; doi:10.1111/jcmm.15806)

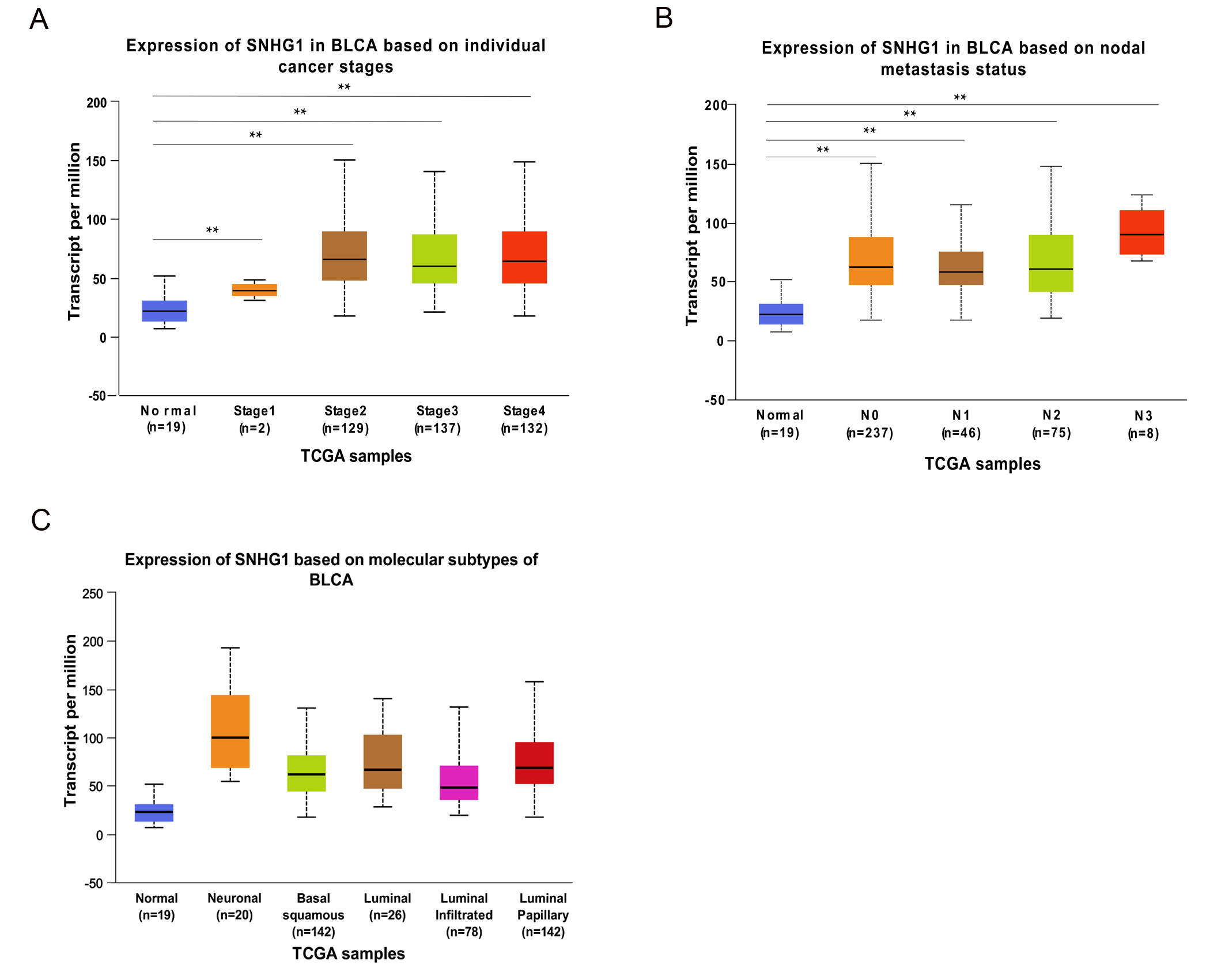

Supplement: Supplementary file 1 — Fig S1 [file JCMM-24-11858-s001.jpg]

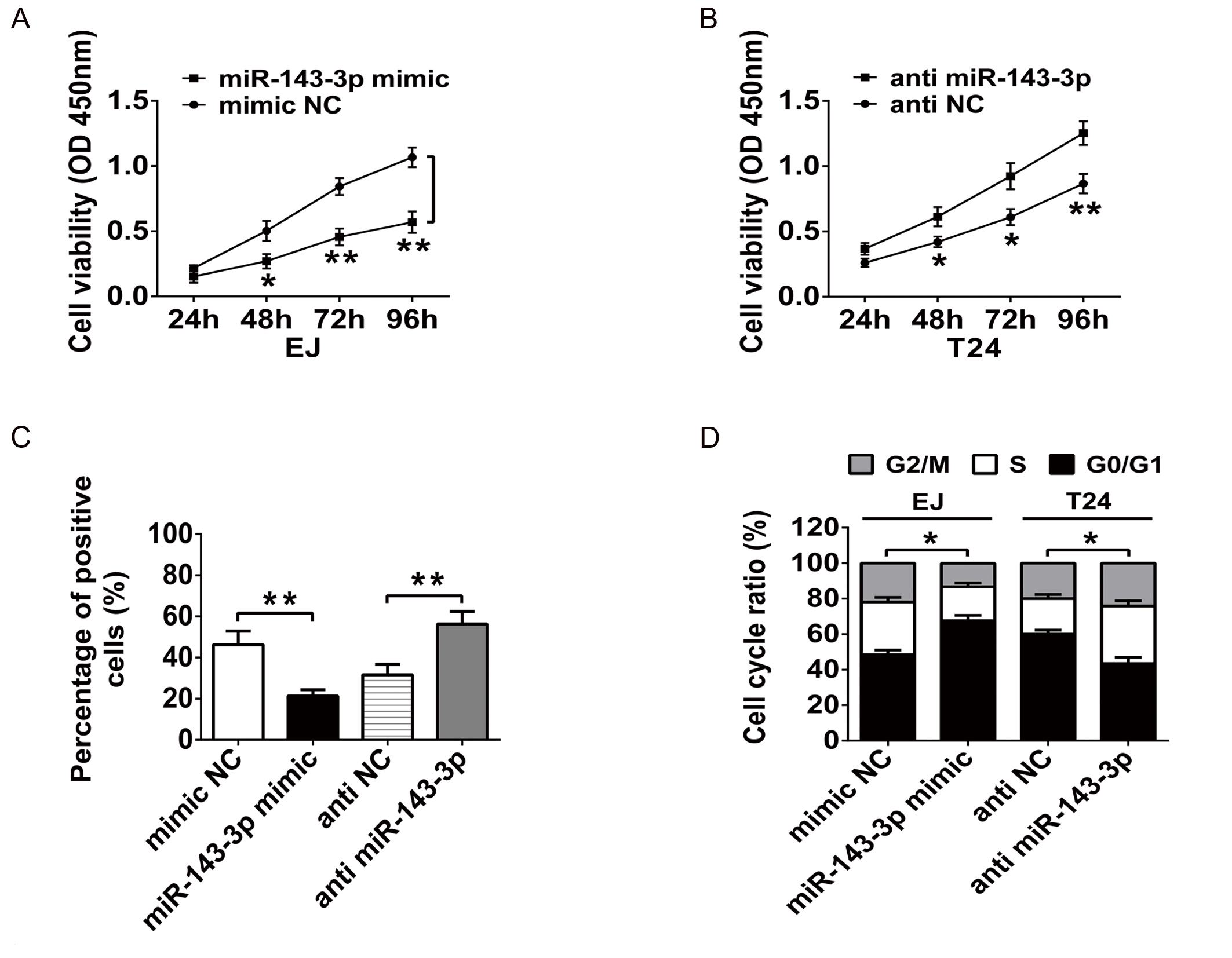

Supplement: Supplementary file 2 — Fig S2 [file JCMM-24-11858-s002.jpg]
