# Supplementary material for: The long non‐coding RNA SNHG1 promotes bladder cancer progression by interacting with miR‐143‐3p and EZH2
Source: J Cell Mol Med. 2020 Sep 4;24(20):11858–73. doi: 10.1111/jcmm.15806 (PMC7578868; doi:10.1111/jcmm.15806)
Supplement: Supplementary file 3 — Table S1 [file JCMM-24-11858-s003.docx]

**Supplementary table S1:**

| **qPCR primers** | |  |
| --- | --- | --- |
| GENE | **Forward Primer** | **Reverse Primer** |
| SNHG1 | ACAAGGGCTTGGTTTTGTTAC | AGTGCCTGAGTTTGGGTTCT |
| HK2 | GAGCCACCACTCACCCTACT | CATTGTCCGTTACTTTCACCCA |
| CDH1 | TGCTCACATTTCCCAACTCCT | GCTCTGTCACCTTCAGCCATC |
| GAPDH | TCAAGAAGGTGGTGAAGCAG | CGTCAAAGGTGGAGGAGTG |
| ASAP3 | CAGAGAGGAACACCTGAGCC | TGGTCAGGCAGCCATACTTG |
| NUAK2 | CATGGAGTCGCTGGTTTTCG | GTCTCCAGGAACTCGTAGCG |
| ZEB1 | CGCAGTCTGGGTGTAATCGT | TGCCGTATCTGTGGTCGTG |
| CTNND1 | CAGAGCGTTACCAAGAGGCA | CCCTCTGGAGAACCACTCATC |
| KRAS | TCCCAGGTGCGGGAGAG | TCAAGGCACTCTTGCCTACG |
| FRS2 | CAAGATTCGGGCCGGAGAG | GAGCCCCTACTTGGAGCAGA |
| KLF5 | AGGACGTTGGCGTTTACGTG | GAGGTTTCGGGTGGACTCCT |
| MAPK7 | AATCTGTCTACGTGGTCCTGG | AGTACTGATGTTCAGCGGGC |
| LASP1 | CGAGAAGAAGCCCTACTGCAA | CTGCCACTACGCTGAAACCT |
| MSI2 | ATATGGAGGCAAATGGGAGCC | GAACGTGACGAAACCGAAGC |
| DNMT3A | GGAGAACTGCAGGGCGAAG | GTGTTGAGCCCTCTGGTGAA |
| WNT1 | CTTCGGCAAGATCGTCAACC | CACACGTGCAGGATTCGATG |
| STAB1 | CCAGGTTGGAAAGTGGAATCC | GGGGCAACTGTGTAACTGAAT |
| MYT1 | ACTCCAGGCACCGAAGTTTAC | AGAGGCGTCCTTCACCTCA |
| CDKN1A | TGTCCGTCAGAACCCATGC | AAAGTCGAAGTTCCATCGCTC |
| CDKN2B | ACTAGTGGAGAAGGTGCGAC | CCATCATCATGACCTGGATCG |
| CDKN1C | ATTTAGAGCCCAAAGAGCCCC | CGGTTGCTGCTACATGAACG |
| KLF2 | CTTCTCTCCCACCGGGTCTA | TAGCCCAAAAATGCCCACCT |
| RUNX3 | ATTCATTCATTCCCCGTGGC | GGTCCCACAGGAATGTCTAGC |
| CNR1 | CTCCGTAGTCAGTGGGGGATA | CAGGGGGCAATCCCTTCG |
| **primers for ChIP assay** | |  |
|  | Forward Primer | Reverse Primer |
| CDH1 | TGAGACAGGAGAGTCTCTTGAACC | CCACTGAGCTAGCAGCCTAATT |

**Sequences for siRNAs**

| SNHG1 #1 | GCUUGUUGCUAUAUACCAUUG | AUGGUAUAUAGCAACAAGCUG |
| --- | --- | --- |
| SNHG1 #2 | GGAGGACAUCAGAAGGUGAAU | UCACCUUCUGAUGUCCUCCAA |
| HK2 | AUGACUUUGGCAAAGUGAGGA | CUCACUUUGCCAAAGUCAUGC |
| CDH1 | GCCGAGAGCUACACGUUCACG | UGAACGUGUAGCUCUCGGCGU |
| EZH2 | AGCAAUAGAUGCUUUUUGGAG | CCAAAAAGCAUCUAUUGCUGG |
| si-Control | CGAATTACTGTGAAAGTCAAT | ATTGACTTTCACAGTAATTCG |

**SNHG1-shRNA #1 sequences cloned into pENTR™/U6 vector**

**Forward Primer:** CACCGGAGCCAATGAAACAGCAGTTCGAAAACTGCTGTTTCATTGGCTCC

**Reverse Primer:**

AAAAGGAGCCAATGAAACAGCAGTTTTCGAACTGCTGTTTCATTGGCTCC

**SNHG1-shRNA #2 sequences cloned into pENTR™/U6 vector**

**Forward Primer:**

CACCGCAGTTGAGGGTTTGCTGTGTCGAAACACAGCAAACCCTCAACTGC

**Reverse Primer:**

AAAAGCAGTTGAGGGTTTGCTGTGTTTCGACACAGCAAACCCTCAACTGC

**SNHG1 overexpressing pcDNA3.1 (+) vectors**

**Forward Primer:**

GGGGTACCGTTCGCTCATTTTTCTACTGCTCGTG

**Reverse Primer:**

CGGGATCCATGTAATCAATCATTTTATTATTTTCATC
